# Role of Clinical Insight at First Month in Predicting Relapse at the Year in First Episode of Psychosis (FEP) Patients

**DOI:** 10.3390/jcm12134261

**Published:** 2023-06-25

**Authors:** Ilias I. Vlachos, Mirjana Selakovic, Irene Ralli, Alexandros Hatzimanolis, Lida-Alkisti Xenaki, Stefanos Dimitrakopoulos, Rigas-Filippos Soldatos, Stefania Foteli, Nikos Nianiakas, Ioannis Kosteletos, Pentagiotissa Stefanatou, Angeliki-Aikaterini Ntigrintaki, Theoni-Fani Triantafyllou, Marina Voulgaraki, Vassiliki Ermiliou, Leonidas Mantonakis, Konstantinos Kollias, Nikos C. Stefanis

**Affiliations:** 1 1st Department of Psychiatry, National and Kapodistrian University of Athens, Eginition Hospital, 11528 Athens, Greecestefania.fotelis@gmail.com (S.F.); kollias@med.uoa.gr (K.K.);; 2 Department of Psychiatry, Sismanoglion General Hospital, 15126 Attica, Greece; 3 Psychiatric Clinic, 414 Military Hospital of Athens, 15236 Palea Penteli, Greece

**Keywords:** FEP patients, insight, relapse, prediction

## Abstract

Introduction: Clinical insight constitutes a useful marker of the progress and outcome of the First Episode of Psychosis (FEP), and lack of insight has been associated with more severe psychopathology, treatment non-adherence, and rehospitalization/relapse. In this study, we aimed to further investigate the possible role of insight as a predictor of relapse, its relation to diagnosis, and other parameters of positive psychotic symptomatology (delusions, hallucinations, and suspiciousness). Methods: The Athens FEP study employed a prospective, longitudinal cohort design in which consecutive newly diagnosed patients with psychosis were interviewed and asked to voluntarily participate after completing informed consent. A total of 88/225 patients were examined at three different time points (baseline, month, and year). Their scores in the relevant items of the Positive and Negative Syndrome Scale (PANSS) were compared (G12 for insight, P1 for delusions, P3 for hallucinations, and P6 for suspiciousness), and they were further associated to diagnosis and the outcome at the end of the year (remission/relapse). Results: In total, 22/88 patients with relapse at the year had greater scores in G12 for both the month and the year, and this finding was corroborated after adjusting the statistical analysis for demographics, diagnosis, social environment, and depression via multiple logistic regression analysis. Moreover, delusions and suspiciousness were significantly higher in patients diagnosed with non-affective psychosis compared to those diagnosed with affective psychosis (*p* < 0.001) at the first month. Conclusions: Lack of insight at the first month may serve as a predictor of relapse at the year.

## 1. Introduction

Clinical insight has been defined as the ability to be aware of a mental disorder; to understand its social consequences; to understand the specific signs and symptoms that are, in addition, attributed to the disorder; and to comprehend the need for treatment [1,2,3]. Insight seems to fluctuate in the course of psychosis from severe impairment in the First Episode of Psychosis (FEP) to improvement in most of the cases in the months following diagnosis [4], and then again from that point to deterioration later on [5]. Insight has also been more associated with the progress than with the outcome of psychosis, and it seems to correspond to symptom severity [6] and share negative correlations with both the positive and negative symptoms of psychosis [7]. Poor insight may prolong the Duration of Untreated Psychosis (DUP), and is associated with low social functioning according to some researchers [8], while other researchers have found a significant relationship between lack of insight and poor general adjustment [9,10] as well as legally enforced hospitalizations of FEP patients [11,12]. On the other hand, good insight has been related to less severe psychopathology [13] and to increased depressive symptoms as much in FEP patients as in schizophrenia patients, which implies a better awareness of their condition amongst the former [14,15]. Unfortunately, early insight and depression have been associated with suicide attempts and with actual suicides within four years after the first episode, which is a known major cause of mortality for schizophrenia patients [16]. In terms of relapse, recent research has shown contradictory results on whether depressive symptoms may predict relapse after FEP or not [17].

While poor insight at discharge of FEP patients along with a diagnosis of schizophrenia after 6 months predict non-adherence to medication treatment [18,19,20,21], antipsychotic medication seems to exert a positive effect on insight, as the European First Episode Schizophrenia Trial (EUFEST) study has suggested [22]. Additionally, poor insight is considered to negatively affect the service engagement of individuals suffering from psychosis [23], especially when it is observed after the first clinical presentation of a psychotic disorder [12].

Related to medication non-adherence and service engagement are the concepts of psychotic relapse and rehospitalization, where clinical insight seems to exert a crucial role [20]. Studies have shown that lack of insight at hospital discharge after a FEP affects the way that patients understand the seriousness of their illness and the importance of medication adherence, which are factors that influence compliance and quality of life as well as relapse and rehospitalization in the first year [18,24,25,26]. 

G12, an item of the general psychopathology scale of the clinician-rated Positive and Negative Syndrome Scale (PANSS) for Schizophrenia [27], is considered a valid and reliable index of clinical insight across studies [7,15,18,22]. It scores between 1 and 7, with scores equal or greater to 4 defining a lack of insight [28]. Although unidimensional in its nature, G12 does not differ significantly as a valid measure of insight compared to other multidimensional methods [13,29,30]. 

Although the association of insight with relapse still constitutes a topic of dispute [12], studies have shown that insight, as measured by G12, may be a predictor of remission and/or relapse after FEP Recently, our research team has published a paper on a study where lack of baseline insight predicted non-remission in FEP patients after 4 weeks [31]. Moreover, Drake et al. [32] have shown that objective 6-week PANSS insight is more predictive of relapse/rehospitalization at the year compared to the subjective Birchwood Insight Scale, which is, at baseline, more predictive of relapse at the year, though.

### Aims

Given the importance of the role that clinical insight plays in the FEP literature, we have attempted in this paper to further explore the potential role of G12 in predicting relapse. We examined (a.) whether lack of insight improvement predicts relapse at the year; (b.) whether insight at 4 weeks (first month) can be predictive of relapse at the year; (c.) whether positive PANSS items P1 (delusion), P3 (hallucination), and P6 (suspiciousness) relate to (i.) insight, (ii.) diagnosis of non-affective psychosis, and (iii) relapse at the year; (d.) whether insight relates to the degree of depression; and (e) whether depression predicts relapse at the year.

## 2. Materials and Methods

### 2.1. Participants

In Greece, there has been no systematic integration of early intervention services for FEP patients in the national health system. Up until now, Eginition University Hospital has had the only intervention unit in Athens for Early and Long-Term Psychosis Intervention Service, including a network of an emergency unit, an acute inpatient clinic, a day hospital, and outpatient early intervention service. In collaboration with four other hospitals in Athens, which provide standard care, a prospective longitudinal research study named the ”Athens FEP study” took place between 2015 and 2020. This study aimed to investigate the involvement of genetic and environmental determinants on psychosis risk. The 225 FEP patients, 16–45 year olds, who presented at the emergency units of the five psychiatric hospitals across Athens, Greece (Eginition University Hospital, General Hospital of Sismanogleio, SNEN 414-Military Hospital, Atticon University Hospital, General Hospital of Sotiria), were, after completing informed consent, recruited in the Athens FEP study. A large battery of psychometric scales, clinical interviews, and laboratory tests at three timepoints measured the psychopathology of each participant: T1: baseline; T2: first month; and T3: year. The recruitment for the longitudinal Athens FEP study lasted 5 years and was carried out by trained mental health professionals in collaboration with the mental health employees of each hospital [33]. Diagnostic Interview in Psychoses (DIP)—confirmed clinical diagnosis [34]. Inclusion criteria concerned ICD 10 diagnoses of F 1x.5, F 20–29, and F 30–33 in patients with first manifestations of psychosis, who were, furthermore, drug-naive or minimally exposed to antipsychotics (less than 2 weeks on medication).

### 2.2. Psychometric Instruments and Outcomes

Our data were derived from 88 out of the initial 225 patients who completed the examination at three time points (T1, T2, and T3). The year’s dropout rate was high (60.9%), which corresponds with the existing literature. In a review by Kreyenbuhl et al. (2009), 18–67% of patients, with a median rate of 58% in the studies, failed to attend a first outpatient appointment after hospital discharge [35]. Insight was derived from the G12 item of the Positive and Negative Syndrome Scale (PANSS). The Scale was developed by Kay, Opler, and Fiszbein in 1987 [27], and is used to measure and grade symptom severity in schizophrenia. It consists of three subscales and 30 items (7 positive items, 7 negative, and 16 general psychopathology items). Each item is graded from 1 to 7, with 1 representing absence of psychopathology and 7 representing the most severe psychopathology interfering with everyday life and probably requiring hospitalization. The PANSS has been translated and published in 40 countries, and it is frequently used in research aside from its clinical application. In our study, we have used the Greek translation of the scale [36]. We checked the correlation of G12 with other PANSS positive items: P1 (delusions), P3 (hallucinations), and P6 (suspiciousness) at the three different time points in order to further clarify the contradictory results of previous research on their interaction [37]. The use of separate items of the positive subscale of PANSS for research purposes has already been validated by the previous literature [37,38,39]. A score of (>4) in any PANSS item was considered as pathological. Moreover, we compared the scores of P1, P3, and P6 items between the relapsed and the non-relapsed patients. The definition of relapse was based on rehospitalization at the year’s follow-up. Improvement of insight was examined at the first month and at the year’s follow up. The symptoms of depression were examined with the Hamilton Rating Scale for Depression [HAM-D] [40] and sociodemographic variables, and the effect of social environment on FEP (via the Social Environment Assessment Tool—SEAT) [41] was also studied.

### 2.3. Statistical Analysis

Quantitative variables are expressed as mean values (SD) or with median and interquartile range (IQR). Qualitative variables are expressed as absolute and relative frequencies. Chi-square and Fisher’s exact tests were used for the comparison of proportions. Wilcoxon signed rank tests were computed to compare quantitative measurements between two different time points. The McNemar test was used to compare the percentages of insight between two different time points. Students’ *t*-test was used for the comparison of a continuous variable between two groups when the distribution was normal, and the Mann–Whitney test was used when the distribution was not normal. The Kruskal–Wallis test was used for the comparison of continuous variables among all diagnoses. Bonferroni correction was used in order to control for type I error. Spearman correlations coefficients were used to explore the association of two continuous variables. All *p* values reported were two-tailed. The association of insight with relapse was tested using logistic regression analyses and after adjusting for sex, age, migration and SEAT-DEPR. Odds Ratios (OR) with 95% Confidence Intervals (95% CI) were computed from the results of logistic regression analyses. Statistical significance was set at 0.05 and analyses were conducted using SPSS statistical software (version 23.0).

## 3. Results

### 3.1. Characteristics of the Sample

The sample consisted of 88 patients (53 mean and 35 women) with a mean age of 26 years (SD = 8.1). Sample characteristics are shown in Table 1. According to G12, 16.1% of the participants had insight at baseline, 69% at the first month, and 86.9% at the year. The percentage at the year was significantly greater compared to that at baseline (*p* < 0.001) and first month (*p* = 0.004). The percentage of patients with insight at the first month was also significantly greater compared to the one at baseline (*p* < 0.001). Additionally, scores on P1 (delusions), P3, (hallucinations), and P6 (suspiciousness) were significantly improved after 1 year (*p* < 0.001).

### 3.2. Association of Absence of Insight with Delusions, Hallucinations, Suspiciousness and Depression

Subjects with absence of insight at the first month had significantly greater P1, P3, and P6 scores at the first month, too (Table 2). No relation was observed between absence of insight and depression at the first month. Subjects with absence of insight at the year’s follow-up had significantly greater scores of P1, P3, and P6 at the year, too. 

### 3.3. Association of Relapse with Absence of Insight and Clinical Parameters

Table 3 presents association of relapse with demographics, insight, depression, and P1, P6, and P3. A total of 22 out of the 88 (25%) patients had relapsed within the year, a percentage already described by a relevant meta-analysis in FEP patients [42]. Patients with relapse had a greater score on G12 at the first month and at the year, while the median change of G12 from baseline to 1 year was lower in those with relapse. Relapse was significantly lower in participants with insight at the first month and at the year. Furthermore, scores at the year for P1, P6, and P3 were all greater for those with relapse. The degree of change in P1, P6, and P3 from baseline to the year was lower in those with relapse. Depression did not differ significantly between patients with relapse and those without.

Adjusting the analysis for sex, age, migration, diagnosis, and SEAT via multiple logistic regression analysis (Table 4), it was found that G12 at both the first month and at the year was associated with relapse at the year. The presence of insight at the first month and at the year was associated with lower odds for relapse. Depression was not associated with relapse.

### 3.4. Association of Diagnosis with Clinical Parameters

G12, P1, P2, and P3 as well as depression scores according to patients’ diagnosis are presented in Table 5. After Bonferroni correction, it was found that P1 at the first month was significantly higher in patients diagnosed with non-affective psychoses compared to patients diagnosed with affective psychoses (*p* < 0.001) and patients with other diagnoses (*p* = 0.001). P6 at the first month was also significantly higher in patients diagnosed with non-affective-psychoses compared to patients diagnosed with other diagnoses (*p* = 0.010). No significant differences were found in G12 at the first month after Bonferroni correction.

## 4. Discussion

In this paper, we have explored the relation of clinical insight at the first month to parameters of positive psychopathology, differential diagnosis, and relapse at the year in a group of FEP patients in Athens, Greece. In agreement with previous research [6,7,8,9,10], lack of insight, as measured by G12, significantly correlated with parameters of positive symptomatology, such as delusions, hallucinations, and suspiciousness (P1, P3, P6) at the three examination timepoints (baseline, month, year) in our sample. Moreover, insight improved significantly from baseline (16,1%) to the first month (69%) to the year (86,9%), a fact that has also been seen in relevant research on FEP patients [4,43,44]. Delusions, hallucinations, and suspiciousness, as measured by the relevant PANSS items, also improved significantly within the year in accordance with the literature [45]

Patients who eventually relapsed after one year manifested increased lack of insight at the first month and at the year compared to non-relapsed patients, while the median change of G12 from baseline to the year was lower in those who relapsed. In contrast, relapse rates were significantly lower in participants with presence of insight at the first month and at the year, while presence of insight at the first month and at the year were associated with lower odds for relapse. After adjusting the analysis for demographics, diagnosis, social environment (SEAT), and depression via multiple logistic regression analysis, it was found that insight (G12) at the first month was associated with relapse at the year.

This finding is of clinical interest, considering the importance of timing in early intervention for FEP patients. Timing has been stressed in the literature either in terms of reducing DUP or developing specialized treatment facilities for this group of patients in order to prevent the transition of FEP into chronicity [33,46,47]. Early detection of patients at increased risk of relapse due to lack of insight after a month of treatment could constitute a useful tool in the direction of early intervention. Previous research underlined the role of the G12 score at six weeks after baseline as a valid predictor of relapse for the year [32]. In our work, this crucial “window” of intervention is reduced from six weeks to four weeks (first month) after baseline.

In terms of diagnosis, patients with non-affective psychoses scored significantly higher in delusions (P1) and suspicion/persecutory ideas (P6) after one month of treatment compared to patients with affective psychoses and other diagnoses in our sample. This finding is supported by recent research where persecutory delusions were related more to patients with non-affective psychoses (schizophrenic, delusional) compared to guilt and grandiose delusions, which were more characteristic for affective psychoses patients [48]. Moreover, lack of insight at the first month showed tendencies of statistical significance in relation to the diagnosis of non-affective psychoses, but did not retain it after Bonferroni correction. Similarly, research has shown that lack of insight may frequently characterize non-affective psychoses, but, nevertheless, it cannot be easily differentiated between schizophrenia and mania in the acute phase, whereas retrospective awareness of illness seems to be poorer for schizophrenia patients [49,50].

Depression scores at baseline and at the first month did not correlate to insight at baseline and the first month, contrary to what some evidence suggests [14,15,16], and nor did they predict relapse at the year as other research shows [17]. They are, however, in agreement with other evidence showing no stable link between insight and depression, hopelessness, or suicidality [49,51] at baseline and the first month.

The limitations of our study include the high drop-out rate between baseline and the year, which is, however, described in the relevant literature [35]. We hypothesize that lack of insight may play a crucial role in the early disengagement of FEP patients from mental health treatment. Moreover, no measure of cognitive insight, such as the Beck Cognitive Insight Scale (BCIS), was used in our study. Cognitive insight describes the metacognitive, self-reflective ability of the patient towards his/her own beliefs, judgments, and experiences [52], and it could constitute a more integrated approach to insight evaluation if it were combined with awareness of illness that clinical insight expresses.

## 5. Conclusions

Lack of insight at the first month significantly correlated with relapse/rehospitalization at the year in our sample of the Athens FEP study patients. Given the importance of the early intervention window in the first episode of psychosis, our results may prove a useful clinical tool of intervention and prevention for this vulnerable group of patients. In conjunction with this finding, the high disengagement rate of FEP patients, also observed in our sample, highlights the necessity of the implementation of Early Intervention Services for Psychoses (EISP) following the example of other countries [53]. A recent meta-analysis shows that the implementation of EISP programs lowers the disengagement and relapse rates of FEP patients compared to treatment as usual (TAU) [54]. An optimistic perspective for Greece is that ten EISP programs are now being established in the National Health System with funds drawn from the Recovery and Resilience Facility of the European Commission (RFF), and they will hopefully start to operate by the end of 2023 [55].

## Figures and Tables

**Table 1:** Sample characteristics: demographics, diagnosis, relapse rate, and insight scores.

	N (%)
Sex	
Men	53 (60.2)
Women	35 (39.8)
Age, mean (SD)	26 (8.1)
Migration	
No	80 (90.9)
Yes	8 (9.1)
Diagnosis	
Non-affective psychosis	63 (71.6)
Affective psychoses	15 (17.0)
Drug-induced psychoses	2 (2.3)
Brief Psychotic Disorder	8 (9.1)
SEAT-DEPR, median (IQR)	15 (13–17.5)
Relapse	22 (25.0)
G12, median (IQR)	
Baseline	5 (4–6)
Month	3 (1–4)
Year	1 (1–3)
Change from baseline to year	−3 (−5–−2)
Insight based on G12	
Baseline	
Absent	73 (83.9)
Present	14 (16.1)
Month	
Absent	27 (31.0)
Present	60 (69.0)
Year	
Absent	11 (13.1)
Present	73 (86.9)

**Table 2:** Association of insight based on G12 at baseline, month, and year with delusions (P1), suspiciousness (P6), and hallucinations (P3).

	Insight Based on G12 at Baseline			Insight Based on G12 at Month			Insight Based on G12 at Year		
	Absent	Present		Absent	Present		Absent	Present	
	Median (IQR)	Median (IQR)	*p*+	Median (IQR)	Median (IQR)	*p*+	Median (IQR)	Median (IQR)	*p*+
P1									
Baseline	6 (6–7)	6 (5–6)	0.352	6 (6–7)	6 (5,5–7)	0.859	6 (5–6)	6 (6–7)	0.592
Month	3 (2–4)	2.5 (1–3)	0.076	4 (3–5)	3 (2–3)	<0.001	4 (3–5)	3 (2–4)	0.066
Year	1 (1–3)	1.5 (1–2)	0.906	2 (1–3.5)	1 (1–2)	0.141	4 (3–4)	1 (1–2)	<0.001
P6									
Baseline	6 (5–7)	5 (4–6)	0.096	6 (5–7)	6 (5–7)	0.596	5 (5–7)	6 (5–7)	0.865
Month	3 (2–4)	2 (1–3)	0.058	3 (3–4)	2 (1–3)	<0.001	3 (3–4)	3 (1–3)	0.047
Year	1.5 (1–2)	1 (1–2)	0.165	2 (1–3)	1 (1–2)	0.006	4 (2–4)	1 (1–2)	<0.001
P3									
Baseline	3 (2–5)	3.5 (2–5)	0.824	4 (3–5)	3 (1–5)	0.324	3 (3–4)	3 (2–5)	0.973
Month	1 (1–2)	1 (1–1)	0.162	2 (1–3)	1 (1–2)	0.033	2 (1–2)	1 (1–2)	0.157
Year	1 (1–1)	1 (1–1)	0.675	1 (1–1)	1 (1–1)	0.698	1.5 (1–3)	1 (1–1)	0.010

+Mann–Whitney test.

**Table 3:** Association of relapse with demographics, diagnosis, social environment insight, delusions, suspiciousness, hallucinations, and depression.

	Relapse		
	No	Yes	
	N (%)	N (%)	*p*
Sex			
Men	39 (73.6)	14 (26.4)	0.706 +
Women	27 (77.1)	8 (22.9)	
Age, mean (SD)	26.2 (7.4)	25.7 (10.1)	0.832 ‡
Migration			
No	60 (75.0)	20 (25.0)	1.000 +
Yes	6 (75.0)	2 (25.0)	
Diagnosis			
Non-affective psychoses	46 (73.0)	17 (27.0)	0.671 ++
Affective psychoses	11 (73.3)	4 (26.7)	
Other ^1^	9 (90.0)	1 (10.0)	
SEAT-DEPR, median (IQR)	15 (13–17)	16.5 (14–20)	0.073 ‡‡
G12, median (IQR)			
Baseline	5 (4–6)	5 (4–6)	0.643 ‡‡
Month	3 (1–3)	4 (2–5)	0.048 ‡
Year	1 (1–2)	3 (2–4)	<0.001 ‡‡
Change from baseline to year	−3 (−5–−2)	−2 (−2–0)	<0.001 ‡‡
Insight based on G12			
Baseline			
Absent	55 (75.3)	18 (24.7)	1.000 ++
Present	11 (78.6)	3 (21.4)	
Month			
Absent	16 (59.3)	11 (40.7)	0.015 +
Present	50 (83.3)	10 (16.7)	
Year			
Absent	4 (36.4)	7 (63.6)	0.001 ++
Present	62 (84.9)	11 (15.1)	
P1, median (IQR)			
Baseline	6 (6–7)	6 (5–6)	0.176 ‡‡
Month	3 (2–4)	3 (2–4)	0.933 ‡‡
Year	1 (1–2)	4 (1–5)	<0.001 ‡‡
Change from baseline to year	−5 (−5–−3)	−2 (-4–0)	<0.001 ‡‡
P6, median (IQR)			
Baseline	6 (5–7)	6 (5–7)	0.250 ‡‡
Month	3 (1–3)	3 (2–4)	0.061 ‡‡
Year	1 (1–2)	3 (1–4)	0.002 ‡‡
Change from baseline to year	−2 (−4–−1)	−1 (−2–0)	0.030 ‡‡
P3, median (IQR)			
Baseline	3 (2–5)	3 (1–5)	0.493 ‡‡
Month	1 (1–2)	1 (1–2)	0.275 ‡‡
Year	1 (1–1)	1 (1–3)	<0.001 ‡‡
Change from baseline to year	−4 (−5–−3)	−2.5 (−4–−2)	0.010 ‡‡

+ Pearson’s chi square test; ++ Fisher’s exact test; ‡ Student’s t-test; ‡‡ Mann-Whitney test. ^1^ Includes drug-induced psychoses and brief psychotic disorder.

**Table 4:** Results from multiple logistic regression analyses with dependent variable, the relapse and independent the insight.

	OR (95% CI) +	*p*
G12 baseline	0.90 (0.62–1.31)	0.581
G12 first month	1.49 (1.06–2.09)	0.023
G12 year	2.60 (1.55–4.38)	<0.001
Insight based on G12 at baseline		
Absent (reference)		
Present	1.09 (0.24–4.98)	0.911
Insight based on G12 at month		
Absent (reference)		
Present	0.28 (0.09–0.93)	0.037
Insight based on G12 at year		
Absent (reference)		
Present	0.10 (0.02–0.47)	0.003

+ Odds Ratio (95% Confidence Interval) adjusted for sex, age, migration, diagnosis, and SEAT-DEPR.

**Table 5:** Association of diagnosis with insight, delusion, suspiciousness, and depression.

	Diagnosis			*p* Kruskal-Wallis Test
	Non-Affective Psychoses A	Affective Psychoses B	Other ^1^ C	
	Median (IQR)	Median (IQR)	Median (IQR)	
HAM-D				
Baseline	18 (12–25)	15 (7–30)	12 (10–23)	0.385
Month	6 (3–12)	7 (4–9)	5 (2.5–8)	0.671
Year	6 (3–10)	4 (1–6)	2.5 (1–4)	0.135
Change from baseline to year	−11 (−19–−7)	−12 (−22–−4)	−6 (−6–−6)	0.635
P1				
Baseline	6 (6–7)	6 (4–7)	6 (5–6)	0.202
Month	3 (3–4) ^b,c^	2 (2–3) ^a^	1 (1–2) ^a^	<0.001
Year	2 (1–3)	1 (1–2)	1.5 (1–2)	0.439
Change from baseline to year	−4 (−5–−3)	−3 (−5–−3)	−4 (−5–−3)	0.971
P6				
Baseline	6 (5–7)	5 (4–7)	6 (4–7)	0.778
Month	3 (2–4) ^c^	2 (1–3)	1.5 (1–2) ^a^	0.005
Year	2 (1–3)	1 (1–2)	1 (1–2)	0.300
Change from baseline to year	−4 (−5–−2)	−4 (−6–−3)	−4 (−6–−3)	0.633
P3				
Baseline	4 (2–5)	3 (1–5)	3 (1–3)	0.292
Month	1 (1–2)	1 (1–2)	1 (1–1)	0.146
Year	1 (1–1)	1 (1–1)	1 (1–1)	0.425
Change from baseline to year	−2 (−4–−1)	−2 (−4–0)	−1.5 (−2–0)	0.329
G12				
Baseline	5 (4–6)	4 (4–5)	4 (3–6)	0.116
Month	3 (1–4)	2 (1–3)	2 (1–3)	0.049
Year	1 (1–3)	1 (1–2)	1 (1–1)	0.153
Change from baseline to year	−3 (−5–−2)	−3 (−4–−2)	−2.5 (−5–−2)	0.999

^a.b.c^ Significant differences after Bonferroni correction; ^1^ includes drug-induced psychoses and Brief Psychotic Disorders.

## Data Availability

Data available upon request.
